# A Plea for the More Intimate Knowledge of Drugs

**Published:** 1914-02-15

**Authors:** Elmer B. Hosom


### A PLEA FOR THE MORE INTIMATE KNOWLEDGE OF DRUGS.

BY ELMER B. HOSOM, D. D. S.

Read before the Jackson, Mich., Dental Club, Nov. 5, 1913.

It is the desire within the brief scope of this paper to merely touch upon the more serious side of our practice of the "healing art," than which there is none so vastly important, and whose deductions are so little known scientifically among the rank and file of the profession as that of the numerous agents employed for the induction of anaesthesia. That any should doubt this let him but hark back to some of the harrowing scenes of his halcyon (?) days in which he appeared as the central figure unscathed,—perhaps.

The dentist with his remedial sleep producing or paralyzing anaesthetic agents fresh from the pharmacist—if he doesn't make them himself—proceeds with his special brand as a kind of multum in parvo. His technique is faultless and silent and he obtains in the minimal length of time the results he seeks. But does he?

It is well for us at this juncture to note that the heart and mind being correlated deal gently with our bruises because both are capable of much kindness and indulgence. Also, time is a potent factor; as it teaches us to forget the harrowing scenes through which we have passed, and turns them many times to good account. It is true that enormous numbers of anaesthetics are given throughout the land, running into many thousands per annum, and fatalities are proportionately rare. But the writer's suspicion is not abated in the least, for every anaethetist dealing with these powerful agents knows that there are infinitely more fatalities than ever reach the public eye. And this is not all, as every anaesthetist, knows, and I assume that each of us is an anaesthetist so long as his requirements are within the realm of reducing pain by these paralyzing and sleep-producing agents—that, short of death there are a great many more serious after-effects to be reckoned with, such as illness, discomforts, and actual disabilities resulting from the injudicious selection of contra indicated anaesthetics. The employment of anaesthetics by those not skillfully trained in their use, is unfortunate and should be condemned. There is no danger of your essayist making this statement too emphatic. The patient should not escape his full share of criticism, for indeed, it is little short of amazing to see with what impunity and indifference he submits himself to the anaesthetic without inquiring to what extent the administrator is acquainted with the drugs he uses or his dexterity in the control of them. It must be borne in mind, in view of the great power for harm as well as for good that these drugs possess, that safety depends entirely upon the choice of the agent and the operator's skill and quick resourcefulness in combating any unfavorable symptoms that not infrequently arise.

It is the anaesthetist and not the anaesthetic that kills many times, as has been tersely and well said by one authority. As to the truth of this statement in many cases there can be no doubt, but the dangers attendent upon the administration of an anaesthetic in the hands of a skilled administrator is to all intents and purposes practically reduced to a reasonable minimum. In this great field of medical science and art, the alleviation of pain and suffering in the operating chair or on the operating table, the heritage is ours by the right of discovery, but there are no "nevers," and no one can say that there *never* can be a death in the hands of the competent. The chances of such a result are so small in fact as not to incur doubt even among those whose duty calls them daily to attend to this kind of work. According to one writer, these chances are no greater than existed in the pre-anaesthetic days, when patients died from fright before the operation had begun. That such accidents occurred is a matter of record, but that they were extremely rare I question and so do you.

Nearly all the known anaesthetics are tissue poisons. This statement must be true, since any drug which has the power of producing profound sleep, or of paralyzing tissue, will have a decided pathologic reaction of a more or less morbid nature, varying according to the temperament, habits, occupation, and physical state of the patient. They have the power of destroying life if taken into the system in sufficiently strong doses or are administered for a sufficiently long period of time. Their deleterious effects are produced in various ways. One anaesthetic acts upon the muscle fibers of which the heart is composed, gradually softening and weakening them until the whole organ becoming involved is incapable of proper contraction and ceases to beat, another gradually paralyzes that nervous center in the brain by continued action of which the breathing is normally carried out; another affects the vessels through which circulation is carried on; and so on. The knowledge of the action of anaesthetics has been discovered not merely by the years of chemical experience in administration to human subjects, but much more decisively from experiments on the lower animals. In this way we are steadily advancing not only in scientific knowledge of what takes place in the most minute recesses, as it were, of a man's being while he is unconscious, but by the use of new and tried drugs, new and tried methods, and new and tried apparatus, to render the administration of our anaesthetics safe and practicable procedures.

It seems strange that so little of the dangers of anaesthetics is known to the general public, and the writer has often experienced the keenest desire to do something to lift from our lights the proverbial bushel that the true light may shine out on the ramparts of human progress along with other lines of human endeavor with measured, stern and dignified pace.

The dawn of an approaching day brings light upon a new era for us, and, not, like Horace of old, we have not had our hour, but we are having it.

There are those not infrequently met who imagine that the anaesthetist's duty is over when the patient is put to sleep, but as a matter of fact his work is then but actually begun. But even the initial anaesthetic can be well or badly handled, safely or hazardously, bunglingly or tactfully executed. It is for the anaesthetist to judge when and by what method and by what drug—anaesthesia is to be accomplished. He should be alert and on the lookout for every departure on the part of the patient from ordinary health. Any such departure noted may render it entirely necessary to vary the procedure, or even to pronounce it unsafe to proceed at all. Little or none of this knowledge is in the hands of the untrained man, and he it is who often glibly undertakes to "enter in where angels dare not tread."

It may well be imagined, then, with what anxiety the public should regard the wholesale use of anaesthetics by those incapable of coping skillfully with the technique of these serious drugs, and, further, there is due some explanation or apology to the trained man, who realizes the importance of this work, who must feel the keenest anxiety, if not contempt, for those of us who, scientifically untrained, indulge in this work with entire satisfaction to ourselves.

It is not only the general anaesthetics, such as nitrous oxide, ether, chloroform, or somnoform which produce unconsciousness that are dangerous when improperly used, but the same criticism may apply to the careless or ignorant use of the local anaesthetics, bodies which are injected and do not produce unconsciousness, but merely local insusceptibility to pain. Some of these bodies are in themselves capable of producing rapid and alarming results, and they are all of them capable of producing much harm if the strictest "surgical cleanliness" is not observed in the process of their employment.

When a careless or unclean injection is made there may easily follow a local and, possibly also, a general and highly dangerous form of blood poisoning.

A mere working knowledge is not enough, unless a working knowledge is taken to mean a thorough knowledge of the *uses* and *application* of such drugs. When the *use* of a drug is referred to we simply mean that that drug is indicated to produce such anaesthesia as the conditions may demand. And by the same token, when the *application* of a drug is referred to, we simply mean that that drug shall be administered with the best known and approved technique and complete knowledge as to its physiological action.

It is generally conceded that the dental surgeon has reasonable technical skill, which is acquired by years of patient training, circumscribed, as it were, and confined to the human mouth, and I may add with pardonable pride that the charge is fairly well supported. But gentlemen, do we not give as dental surgeons in the acquiring of skill along certain traditional hard and fast lines, too much of our best time and energies to the exclusion of other most important and vital considerations? We are so prone to be hampered by the restricted character of our field to have views which emanate an effervescent confusion and indicision from the four walls within which we carry on our daily practices to the point, I fear, of losing sight of the object itself and of worshiping only the shadow. To be more explicit, we seek quick returns in simple procedures which thought and reflection would entirely overcome and change. This is obvious and need not seriously draw upon your imagination.

As the immortal Daniel Webster once said in one of his famous addresses: "And what does this all amount to any way." Well, I'll tell you. If we demanded remuneration as professional men commensurate with the seriousness of the operations we many times undertake, the undertaking itself would lose much of its levity, not only with the public, but the profession itself as well as the individual operator and patient would quickly appreciate the importance of encouraging higher knowledge and skill by adequate appreciation and compensation.

Technically, in so far as prosthetic art, ceramic art, operative skill, oral hygiene and minor oral surgical operations are concerned we have gone far, challenging admiration scarcely short of wonder in these and various fields because of the knowledge and skill accumulated, perhaps more in the technical than in the scientific departments.

In fact, the scope of dentistry has become so broad and far-reaching that the advent of specializing may be expected in the near future,—if indeed it has not already arrived,—it will be the rule then and not the exception to know our medical science as well as our dental art.

Allow me to prophesy a word of warning—before another decade shall have passed into history judging the future by the past, which is an unerring guide, our work will largely be cut out for us. The public will lead and we shall follow the demand for a stricter specializing of our professional work. This is but and instance, for the air we breathe is surcharged with this ever increasing modern tendency to specialize. Nor is it a fad, it is the evolution of first principles simplified to become more definite.

The wave of human progress in all callings is sweeping us too with an intensity that cannot be turned aside and it will carry us inevitably along to higher attainments in the knowledge of our calling to the good of mankind.

This is the history of human achievement all along the centuries down to the present. Let us then declare ourselves; clear the decks of our fleet and meet without fear or favor these public demands.

With your indulgence I will suggest the manner of clearing the deck of Flag Ship "Anaesthesia." This, you know is a very important ship. In order to steer this "Monitor of Destiny" safely to port our nautical terms shall include, in part, first: a revival of physiology; second, physiological chemistry; third, diagnosis; fourth, surgical cleanliness; fifth, pathology; sixth, restoratives; seventh, prognosis; and lastly, all necessary details.

I leave the subject to you in the hope that a good, lively discussion may follow this very important suggestion of "A plea for the More Intimate Knowledge of Drugs." The writer begs to acknowledge his own short-comings but with an ever increasing desire to learn, and with hoping that he has passed through with you the first lines of that famous Arabian Proverb nor ceasing in energy and initiative until we have passed to the last line, thereof:

"He who knows not, and knows not that he knows not, is a
fool—shun him.
He who knows not, and knows that he knows not, is
simple—teach him.
He who knows, and knows not that he knows, can read and
will learn—learn with him.
He who knows, and knows that he knows, is wise—
learn of him."

DISCUSSION OF DR. HOSOM'S PAPER BY DR. R. O. CURTIS

I wish to congratulate the essayist on the very able paper he has given us tonight, I also want to thank the other members of the society for the way they have responded to the requests of your programme committee for the several parts assigned them.

I must however, differ with the essayist in his contention that "*we should worry.*" We do not need to know anything scarcely about drugs in this great age of patent dope and blue sky.

Dr. Barbour tells us that if we will follow a few very simple directions which he will give us for the paltry sum of $50 we simply can't have any trouble giving nitrous oxide, or avoid getting rich.

Then for local specialist we have the inventor of "sweet air" and the discoverer of "narcola" both of whom fully guarantee results, and I doubt not, would be glad to help us get rich.

If we have an abscess or gum boil, we are urged to use "oxapara." Full directions on the box.

If we have pyorrhoea of the gums we have a wide choice from "*gum ease*" to "*dentinol*" and "*pyorrhocide*" in connection with "*Sal-hepatico*" or "*prunoids.*"

In case we want to extract the teeth, we have an endless list of local anaesthetics, such as "*Miller's*, "*Higgins*," "*Waites*," "*Non Toxo*," "*Non Hurt*," *etc.*, all of which are claimed to be absolutely safe and reliable.

So, really, time spent studying Materia Medica is time wasted, besides it makes you worry.

One-fourth of a grain of cocaine is the maximum dose that should enter the circulation at one time, and 23 drops of a one per cent. solution gives you the one-fourth of a grain of cocaine.

According to the advertisements you can inject a barrel of their patent anaesthetics with no harmful effects.

Who will say, therefore, that ignorance is not bliss?

